# Late Bedtime and Poor Sleep Quality Among University Esports Players: A Cross-Sectional Study

**DOI:** 10.3390/healthcare14183103

**Published:** 2026-09-20

**Authors:** Aysun Yuksel, Hulya Yilmaz Onal, Ayşe Betül Bilen

**Affiliations:** 1 Department of Nutrition and Dietetics, Faculty of Health Sciences, Istanbul Medeniyet University, Istanbul 34862, Turkey; aysun.yuksel@medeniyet.edu.tr (A.Y.); hulya.onal@medeniyet.edu.tr (H.Y.O.); 2 Department of Nutrition and Dietetics, Hamidiye Faculty of Health Sciences, University of Health Sciences, Istanbul 34668, Turkey; 3 Department of Nutrition and Dietetics, Faculty of Health Sciences, Atlas University, Istanbul 34408, Turkey

**Keywords:** esports, sleep quality, PSQI, Mediterranean diet, bedtime, sleep timing

## Abstract

**Highlights:**

**What are the main findings?**

- Nearly one in two university esports players had poor sleep quality, and each one-hour delay in bedtime was associated with 33% higher odds of poor sleep quality.
- Among the evaluated sleep-timing indicators, bedtime showed the most favorable numerical model performance indices, and the association remained evident in sensitivity analyses accounting for the circular nature of clock time, potential measurement coupling within the PSQI, and self-reported sleep duration.

**What are the implications of the main findings?**

- Later bedtime may represent a relevant behavioral correlate of poor sleep quality among university esports players and warrants further investigation in prospective studies.
- Prospective and experimental studies are needed to determine the direction of the association and whether modifying sleep timing improves sleep quality in this population.

**Abstract:**

**Background/Objectives**: Sleep timing is an important dimension of sleep health, yet its association with sleep quality among university esports players remains unclear. This study examined factors associated with poor sleep quality, emphasizing habitual bedtime. **Methods:** This cross-sectional study included 164 university esports players with previous competition experience. Sleep quality was assessed using the Pittsburgh Sleep Quality Index (PSQI). Multivariable logistic regression examined factors associated with poor sleep quality, with bedtime modeled continuously. Sensitivity analyses addressed circular clock time, PSQI measurement coupling, and self-reported sleep duration. **Results:** Poor sleep quality was identified in 50.6% of participants. Self-reported chronic disease (OR = 5.60, 95% CI: 1.72–18.28; *p* = 0.004) and each one-hour delay in bedtime (OR = 1.33, 95% CI: 1.13–1.57; *p* < 0.001) were associated with poor sleep quality after multivariable adjustment. Bedtime showed the most favorable numerical model performance indices among the evaluated sleep-timing indicators. The association with later bedtime persisted in circular analyses, after excluding the PSQI habitual sleep-efficiency component (β = 0.327, 95% CI: 0.165–0.490; *p* < 0.001), and among participants reporting at least 7 h of actual sleep (OR = 1.49, 95% CI: 1.16–1.91; *p* = 0.002). **Conclusions:** Poor sleep quality was common among university esports players. Later bedtime was consistently associated with poor sleep quality, including among those reporting at least 7 h of actual sleep. Habitual bedtime may warrant consideration alongside sleep duration when assessing sleep health in this population. Longitudinal studies are needed to establish directionality.

## 1. Introduction

Sleep is a fundamental biological requirement for maintaining physical and mental health, preserving cognitive function, and supporting daytime performance. Insufficient or disrupted sleep has been associated with adverse outcomes for cognitive function, mental health, and cardiometabolic health [1,2]. Yet healthy sleep cannot be defined by total sleep duration alone. Sleep quality, timing, regularity, continuity, and functioning during wakefulness are also core components of sleep health, alongside duration [1,2]. Seven to nine hours per night is generally considered appropriate for young adults [3], though meeting this recommended duration alone does not guarantee optimal sleep health. Later sleep timing and greater variability in sleep schedules have been associated with adverse health outcomes, suggesting that when and how consistently sleep occurs may be relevant in addition to total sleep duration [4]. Habitual bedtime should, however, be distinguished from related aspects of sleep timing. Bedtime is a behavioral clock-time measure, whereas chronotype reflects a preference for earlier or later sleep–wake activity [5]. Circadian phase refers to the timing of the endogenous circadian system and is typically assessed using physiological markers such as dim light melatonin onset rather than inferred directly from reported bedtime [6]. Sleep regularity and social jetlag describe variability in sleep timing across days rather than bedtime itself [4]. In the present study, bedtime was therefore considered a behavioral indicator of habitual sleep timing.

Electronic sports (esports) is a performance field in which video games are played individually or as part of a team, within structured rules and competitive organizations. Unlike recreational gaming, esports involves competitive elements such as systematic training, in-team coordination, performance evaluation, and tournament participation. Esports performance requires the simultaneous use of cognitive and psychomotor skills, including sustained attention, rapid information processing, working memory, decision-making, visual–motor speed, and fine motor coordination [5]. Adequate, good-quality sleep therefore matters not only for players’ general health but for sustaining the cognitive functions competition demands.

At the same time, certain features of the esports environment may be associated with less favorable sleep behaviors. Long and variable training hours, matches that extend into the evening or night, online activity with players across different time zones, performance pressure, and cognitive arousal that continues after a game ends have been associated with delayed sleep onset [7,8]. Prolonged nighttime screen exposure, irregular daily schedules, and caffeinated beverage consumption are also considered among the behavioral factors associated with sleep timing and sleep onset [7,8,9]. For university-level esports players, class schedules, exam periods, academic responsibilities, and the time demands of social life can add to these factors. Similar competing demands have been described in collegiate student-athletes, whose sleep may be constrained by academic, athletic, and social responsibilities as well as variable training and competition schedules [10]. Recent studies have also emphasized that sleep quality in student-athletes should be considered within a broader lifestyle context that includes chronotype and dietary behaviors [5,11]. Although university esports players differ from traditional student-athletes in the nature of their performance demands, both groups must balance competitive participation with academic responsibilities. This combination of academic and performance-related demands may make habitual bedtime particularly relevant in university esports players, as many esports-related activities and exposures occur during the evening and may extend close to the intended sleep period. Habitual bedtime therefore provides a behavioral measure of when players transition from these late-day activities to their main sleep period.

Research conducted in general gaming populations shows that intense or problematic gaming behavior is associated with poor subjective sleep quality, short sleep duration, late bedtime, insomnia symptoms, and daytime sleepiness [9,12,13]. However, the direct transfer of findings obtained from general video game players to esports players may be limited. Esports participation differs from recreational gaming in terms of competitive demands, performance expectations, and competition-related stress. Studies examining esports players directly have reported late sleep onset and wake times, insufficient sleep duration, poor sleep efficiency, and subjective sleep problems [13,14,15,16]. A multinational pilot study of professional players found markedly delayed sleep timing and prolonged nighttime wakefulness [14], while a study comparing Korean professional players with age-matched controls similarly found that esports players went to bed later and reported lower subjective sleep quality [15]. A substantial portion of this evidence, however, comes from small samples, professional teams, or specific game genres. Professional esports players typically operate within performance-centered training environments, whereas university esports players must balance competitive gaming with academic schedules and other demands of student life. Sleep patterns reported in professional teams may therefore not fully reflect the behavioral context of university-level esports players.

Evidence on the prevalence of poor sleep quality and its associated health and lifestyle factors among university esports players remains limited. Existing studies have largely focused on total sleep duration or general sleep characteristics, and the relationship between sleep quality and different sleep timing indicators, such as bedtime, wake time, and mid-sleep time, has not been sufficiently examined. Examining habitual bedtime as a continuous predictor allows its association with poor sleep quality to be evaluated across the observed range of bedtime, without imposing an arbitrary cut-off to define “early” or “late” bedtime [4]. Yet sleep timing represents a dimension of sleep health different from total sleep duration, and this may carry particular importance for esports players because of training and competition schedules extending into late hours [1,4,12]. Determining whether the relationship between sleep timing and sleep quality persists among players reporting at least 7 h of actual sleep may also help clarify whether sleep timing provides relevant information beyond meeting the minimum recommended sleep duration.

This study aimed to determine the frequency of poor sleep quality and its associated factors among university esports players, with particular attention to habitual sleep timing. We examined bedtime, wake-up time, and mid-sleep time in relation to poor sleep quality and assessed whether the association between bedtime and sleep quality remained evident among players reporting at least 7 h of self-reported actual sleep. We hypothesized that later habitual bedtime would be associated with greater odds of poor sleep quality after adjustment for relevant demographic, health, dietary, and gaming-related factors.

## 2. Materials and Methods

### 2.1. Study Design and Participants

This cross-sectional study was conducted in Istanbul, Türkiye, between February and April 2025. Participants were recruited using convenience sampling through direct face-to-face contact in university settings. The data collection team visited campuses and student dormitories at nine universities and reached potential participants through esports-related activities and settings. Participants from seven of the nine universities were enrolled in the study. No formal contact was made with university administrations. Although participants were reached in settings associated with university esports activities, formal membership in a university esports club or inclusion on an official team roster was not independently verified. Data were collected face-to-face using self-administered questionnaires.

A total of 193 university students engaged in esports were approached, of whom 186 agreed to participate and completed the questionnaire. Prespecified eligibility criteria included being at least 18 years of age, being a university student engaged in esports, having previously participated in an esports competition, and providing written informed consent. Of the 186 respondents, 19 were excluded because they had not previously participated in an esports competition, one was excluded because of a self-reported diagnosed psychiatric disorder, and two were excluded because of incomplete or invalid questionnaire data. The final analytic sample therefore comprised 164 university esports players with previous competition experience and complete data for the primary outcome and variables included in the multivariable analyses.

Participants reporting a previous psychiatric diagnosis were excluded according to eligibility criteria prespecified before data collection, given the well-established relationship between psychiatric conditions and subjective sleep disturbances. This criterion was based on self-reported previous diagnosis. No additional screening instrument was used to assess current psychological symptoms or undiagnosed psychiatric conditions; therefore, participants with undiagnosed symptoms or conditions may have been included.

The study was conducted in accordance with the Declaration of Helsinki and was approved by the Hamidiye Scientific Research Ethics Committee of the University of Health Sciences (Protocol No. 2024/6; Decision No. 24/359). Written informed consent was obtained from all participants before data collection. All data were anonymized prior to analysis, and participant confidentiality was maintained throughout the study.

### 2.2. Measurements and Instruments

**Pittsburgh Sleep Quality Index (PSQI):** Sleep quality was assessed using the Pittsburgh Sleep Quality Index (PSQI), a validated self-report questionnaire evaluating sleep quality during the previous month [17]. The PSQI consists of 19 self-rated items generating seven component scores and a global score ranging from 0 to 21, with higher scores indicating poorer sleep quality. Consistent with the original recommendation, participants with a global PSQI score > 5 were classified as having poor sleep quality. In addition to the PSQI global score, habitual bedtime, wake-up time, and sleep duration reported in the PSQI were extracted for analyses of sleep timing. The Turkish version of the PSQI has demonstrated acceptable validity and reliability [18].

**Mediterranean Diet Adherence Screener (MEDAS):** Diet quality was assessed using the 14-item Mediterranean Diet Adherence Screener (MEDAS) [19]. The total score ranges from 0 to 14, with higher scores indicating greater adherence to the Mediterranean diet. For descriptive purposes, participants were additionally classified into adherence categories. The Turkish version of the MEDAS has been validated previously [20].

**Other Variables:** Sociodemographic and lifestyle variables included age, sex, self-reported chronic disease status, smoking, alcohol consumption, body mass index (BMI), gaming-related characteristics (years in esports, weekly gaming frequency, daily gaming duration, and number of competitions won), dietary habits (meal frequency, snack frequency, breakfast skipping, first meal before 09:00, eating after 21:00, dietary supplement use, and daily water intake). Chronic disease was recorded as a self-reported binary variable (yes/no).

**Sleep Timing Variables:** Habitual bedtime and wake-up time were obtained from the PSQI. Because bedtime extended across midnight, clock times were transformed into a continuous late-night scale for regression analyses by expressing post-midnight times as values greater than 24:00 (e.g., 01:00 = 25.00, 03:30 = 27.50). This transformation preserved the chronological ordering of sleep onset while avoiding the discontinuity introduced by midnight. Mid-sleep time was calculated as the midpoint between bedtime and wake-up time. The interval between habitual bedtime and wake-up time was calculated for descriptive purposes. For the duration-restricted sensitivity analysis, the PSQI sleep-duration component was used; a component score of 0, corresponding to at least 7 h of self-reported actual sleep, was considered to indicate meeting the minimum recommended sleep duration for adults.

### 2.3. Statistical Analysis

Statistical analyses were performed using IBM SPSS Statistics version 22 (IBM Corp., Armonk, NY, USA) and Python version 3.13.5 (Python Software Foundation, Wilmington, DE, USA), with the statsmodels (version 0.14.6), SciPy (version 1.17.0), pandas (version 2.2.3), and NumPy (version 2.3.5) packages used for additional sensitivity analyses, circular statistical procedures, and model diagnostics conducted during revision. Continuous variables are presented as mean ± standard deviation (SD) or median (interquartile range, IQR), depending on their distribution, and categorical variables as frequencies and percentages. Comparisons between participants with good and poor sleep quality were performed using the Mann–Whitney U test for continuous variables and the chi-square test for categorical variables. Effect sizes are reported as rank-biserial correlation for Mann–Whitney U tests and Cramer’s V for categorical comparisons. A two-sided *p*-value < 0.05 was considered statistically significant.

A binary logistic regression model was constructed with poor sleep quality (PSQI > 5) as the dependent variable. Because habitual bedtime crossed midnight, clock times were transformed to a continuous late-night scale by adding 24 h to times recorded between 00:00 and 11:59. Bedtime was therefore modeled as a continuous predictor (odds ratio per one-hour delay) rather than categorized using arbitrary cut-off values. The assumption of linearity for bedtime on the logit scale was evaluated by adding a quadratic bedtime term to the adjusted model and comparing the linear and quadratic specifications using a likelihood-ratio test and Akaike information criterion (AIC). To explore the relative performance of different sleep-timing metrics, three separate adjusted logistic regression models were fitted using bedtime, wake-up time, or mid-sleep time as the continuous timing predictor while keeping all other covariates unchanged. Model performance was descriptively compared using the area under the receiver operating characteristic curve (AUC), Nagelkerke’s R^2^, Akaike information criterion (AIC), and Bayesian information criterion (BIC).

The relationship between bedtime and the continuous PSQI global score was additionally visualized using locally weighted scatterplot smoothing (LOWESS) curves in the overall sample and among participants with a PSQI sleep-duration component score of 0, corresponding to at least 7 h of self-reported actual sleep.

The recoded linear bedtime variable was used in the primary multivariable model because it provides a directly interpretable estimate of the association per one-hour delay and permits adjustment for multiple covariates within the logistic regression framework. Because clock time is inherently circular and linear recoding across midnight may not fully preserve its cyclical structure, circular statistical methods were additionally applied as a sensitivity analysis to evaluate the robustness of the bedtime association without relying on the linear representation of clock time. Clock times were converted to angular values on a 24 h circle (θ = 2π × hour/24, radians). Circular means and mean resultant lengths (R) were calculated for each sleep-quality group. After confirming similar angular concentration between groups, circular mean bedtimes were compared using the Watson–Williams test. Mardia’s circular-linear correlation coefficient was calculated to assess the association between circular bedtime and the continuous PSQI global score, with statistical significance evaluated using the asymptotic χ^2^ distribution with two degrees of freedom.

As a further sensitivity analysis, the adjusted logistic regression model was repeated among participants with a PSQI sleep-duration component score of 0, corresponding to at least 7 h of self-reported actual sleep, to examine whether the association between bedtime and poor sleep quality remained evident among participants meeting the minimum recommended sleep duration for adults [1,3].

Model estimates are presented as crude and adjusted odds ratios (ORs) with 95% confidence intervals (CIs). Covariates were selected using a combination of theoretical considerations, previous literature, and observed associations in the study sample. Age, sex, BMI, MEDAS score, eating after 21:00, and daily gaming duration were identified before multivariable modeling to represent demographic, anthropometric, dietary, and gaming-related factors considered relevant to sleep quality and sleep timing. Chronic disease status was additionally included based on both its observed univariable association with poor sleep quality and its clinical relevance. All selected covariates were retained in the adjusted model irrespective of their statistical significance in the final model. Because smoking and alcohol use are plausible correlates of sleep quality, an additional sensitivity model further adjusted the primary multivariable model for smoking and alcohol use. Given the high prevalence of poor sleep quality in the sample, a modified Poisson regression model with robust variance estimation was additionally fitted using the same covariates as the primary logistic regression model to estimate adjusted prevalence ratios (aPRs) and 95% CIs. Self-reported sleep duration was not included as a covariate in the primary multivariable model because sleep duration is itself one of the seven components contributing to the PSQI global score, which could introduce part–whole coupling. To address potential measurement coupling between sleep-timing variables and the PSQI global score, an additional sensitivity analysis was performed after removing the habitual sleep-efficiency component (Component 4), which is partly calculated using reported bedtime and getting-up time. A modified continuous PSQI score was calculated by subtracting Component 4 from the global PSQI score. Because the conventional PSQI > 5 threshold is validated for the original seven-component global score, no diagnostic cut-off was applied to this modified score. The association between bedtime and the modified continuous PSQI score was examined using multivariable linear regression with the same covariates as the primary adjusted model.

Model fit was evaluated using the Hosmer–Lemeshow goodness-of-fit test and Nagelkerke’s R^2^, whereas multicollinearity was assessed using variance inflation factors (VIFs). Model discrimination was evaluated using receiver operating characteristic (ROC) curves and the corresponding area under the curve (AUC). Model calibration was assessed using the Brier score and calibration plots. Because model performance was evaluated in the same sample used for model estimation, AUC, calibration, and related performance measures were considered apparent, exploratory measures rather than estimates of internally or externally validated predictive performance.

## 3. Results

Table 1 summarizes the baseline characteristics of the analytic sample (*n* = 164). Overall, 50.61% of participants had poor sleep quality, the mean PSQI score was 5.88 ± 2.69, and median bedtime was 03:00 (IQR: 01:00–04:30). Only 24 participants (14.6%) reported bedtimes at or before midnight. The distribution of the most frequently reported esports game titles is presented in the Supplementary Materials (Figure S1).

Sleep timing was markedly delayed in this sample. Bedtime ranged from 20:00 to 08:30, and the median mid-sleep time (the midpoint between bedtime and wake-up time) was 06:30 (IQR 05:00–08:30).

As shown in Table 2, participants with poor sleep quality had a higher proportion of chronic disease (*p* < 0.001), a shorter bedtime-to-wake-up interval (*p* = 0.003), and a higher frequency of eating after 21:00 (*p* = 0.037). Bedtime also differed significantly between sleep-quality groups (median 02:00 [IQR 00:30–03:00] for good sleepers vs. 03:30 [IQR 02:00–05:00] for poor sleepers; Mann–Whitney U, *p* < 0.001, rank-biserial r = 0.352), with participants reporting poor sleep quality having later bedtimes. Age, BMI, MEDAS score, breakfast skipping, first meal before 09:00, number of main meals, number of snacks, and daily gaming duration did not differ significantly between groups.

Table 3 presents logistic regression results for poor sleep quality (PSQI > 5), modeling bedtime as a continuous predictor (per hour, on the recoded late-night scale) rather than as categorical groups. In the adjusted model, chronic disease was independently associated with poor sleep quality (OR = 5.60, 95% CI: 1.72–18.28, *p* = 0.004). Each one-hour delay in bedtime was associated with 33% higher odds of poor sleep quality (OR = 1.33, 95% CI: 1.13–1.57, *p* < 0.001). There was no evidence that a quadratic specification improved model fit over the linear bedtime specification (quadratic term *p* = 0.247; likelihood-ratio test *p* = 0.231), supporting retention of bedtime as a linear continuous predictor. In contrast, age, sex, BMI, MEDAS score, eating after 21:00, and daily gaming hours were not independently associated with poor sleep quality in the adjusted model. The multivariable model showed acceptable calibration according to the Hosmer–Lemeshow test (χ^2^ = 9.19, *p* = 0.326), explained a modest proportion of the variance (Nagelkerke R^2^ = 0.245), and showed no evidence of multicollinearity (all VIFs < 1.14).

**Figure 1:**
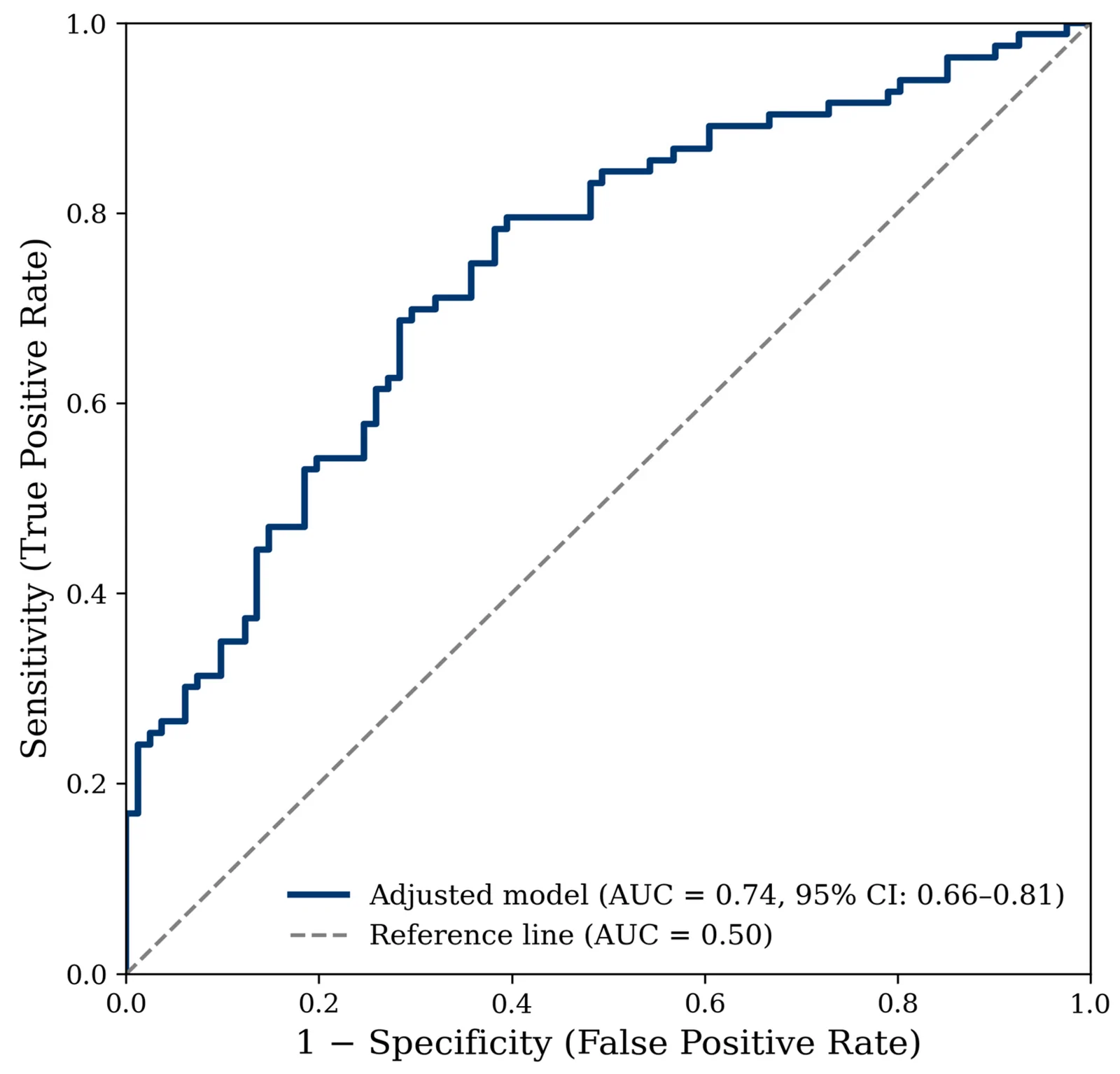
Receiver operating characteristic (ROC) curve for the adjusted multivariable logistic regression model presented in Table 3. The dashed diagonal line represents a non-discriminating model (AUC = 0.50).

**Figure 2:**
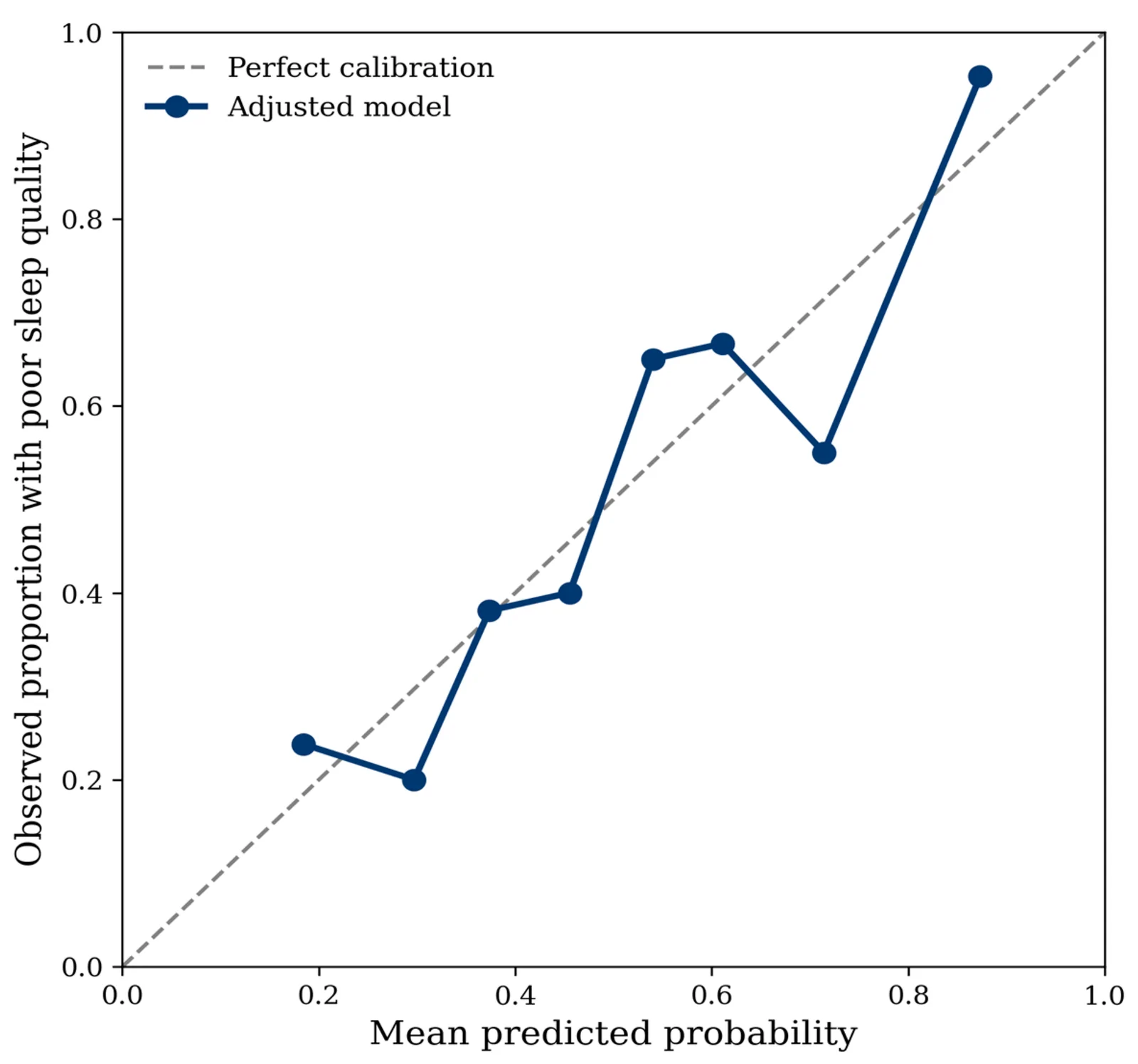
Calibration plot for the adjusted multivariable logistic regression model presented in Table 3. Participants were grouped into octiles of predicted probability; the mean predicted probability in each group is plotted against the observed proportion with poor sleep quality. The dashed diagonal line represents perfect calibration.

The model demonstrated acceptable discrimination (AUC = 0.74, 95% CI: 0.66–0.81; Figure 1) and acceptable overall calibration (Hosmer–Lemeshow *p* = 0.326; Brier score = 0.20; Figure 2). These performance estimates represent apparent, exploratory performance in the derivation sample and should not be interpreted as validated predictive performance.

**Figure 3:**
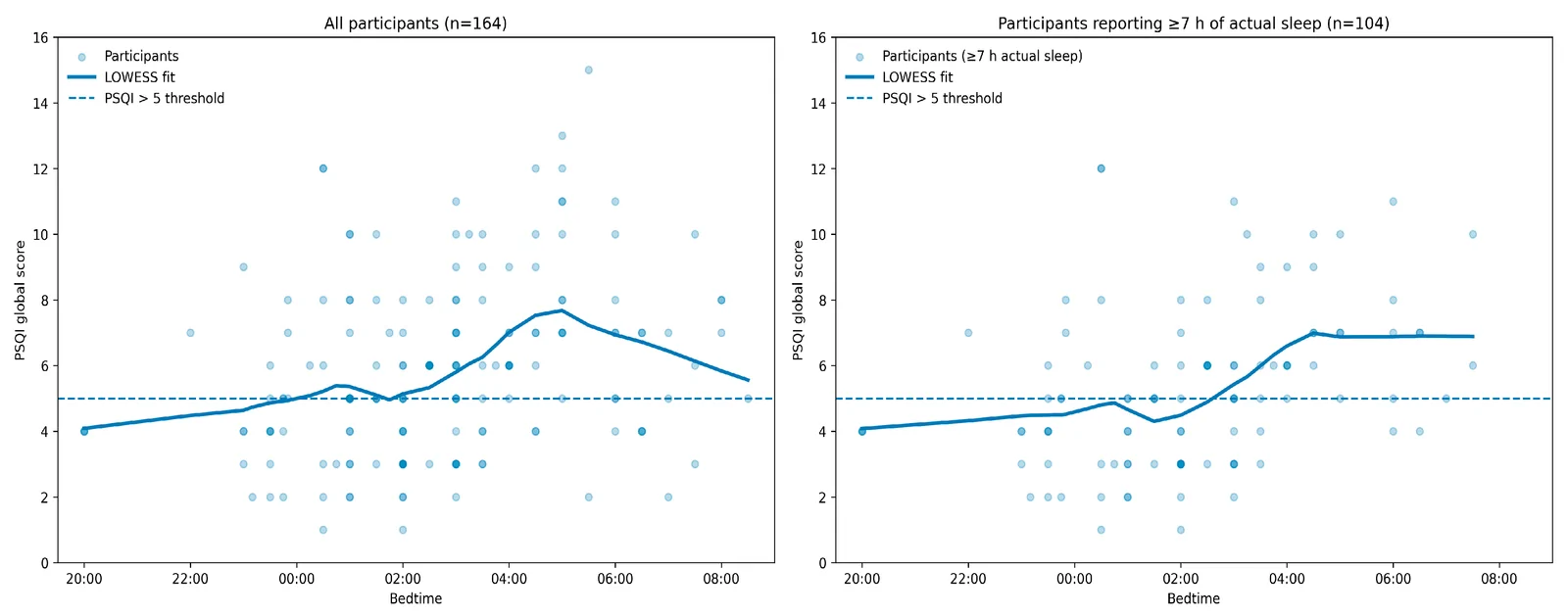
LOWESS-smoothed relationship between habitual bedtime and PSQI global score in the full sample (*n* = 164) and among participants with a PSQI sleep-duration component score of 0, corresponding to at least 7 h of self-reported actual sleep (*n* = 104). The dashed horizontal line indicates the PSQI > 5 threshold for poor sleep quality.

Figure 3 illustrates the relationship between bedtime and the PSQI global score using locally weighted scatterplot smoothing (LOWESS). In the overall sample, progressively later bedtimes were associated with higher PSQI scores. A similar visual pattern was observed among participants reporting at least 7 h of self-reported actual sleep. The smooth curves indicated a gradual increase in PSQI scores across later bedtimes rather than an abrupt threshold.

To explore the relative performance of different sleep-timing metrics, three separate adjusted logistic regression models were compared, each including the same covariates (age, sex, BMI, chronic disease, MEDAS score, eating after 21:00, and daily gaming hours) but substituting a single continuous timing predictor: bedtime, wake-up time, or mid-sleep time. As shown in Table 4, both later bedtime and later mid-sleep time were significantly associated with poor sleep quality, whereas wake-up time was not. Among the evaluated timing indicators, the bedtime model showed the most favorable numerical model performance indices in this sample, with the lowest AIC and BIC and the highest AUC and Nagelkerke R^2^. However, these numerical differences were not formally tested for statistical significance.

As a sensitivity analysis, bedtime was additionally analyzed using circular statistics to account for its cyclical nature (i.e., clock time wraps around midnight) without relying on the linear late-night recoding used in the primary analysis. The mean resultant lengths were similar for the two groups (R = 0.81 for good sleepers, *n* = 81; R = 0.85 for poor sleepers, *n* = 83), supporting the assumption of approximately equal angular concentration required for the Watson–Williams test. The circular mean bedtime was 01:59 for participants with good sleep quality and 03:27 for participants with poor sleep quality. A Watson–Williams test confirmed a statistically significant difference in circular mean bedtime between groups (F(1, 162) = 15.80, *p* < 0.001). Mardia’s circular-linear correlation coefficient between bedtime and the continuous PSQI global score was also significant (R^2^ = 0.093, χ^2^(2) = 15.31, *p* < 0.001). These findings confirmed that the association between later bedtime and poorer sleep quality was also evident when bedtime was represented as a circular variable. In an additional sensitivity analysis further adjusting the primary multivariable model for smoking and alcohol use, the association between later bedtime and poor sleep quality remained essentially unchanged (OR = 1.32, 95% CI: 1.12–1.56, *p* = 0.001). Smoking and alcohol use were not independently associated with poor sleep quality in this expanded model (both *p* > 0.05). Given the high prevalence of poor sleep quality, the primary analysis was additionally examined using modified Poisson regression with robust variance estimation. Each one-hour delay in bedtime remained associated with a higher prevalence of poor sleep quality (aPR = 1.13, 95% CI: 1.06–1.21, *p* < 0.001).

To address potential measurement coupling between bedtime and the PSQI global score, an additional sensitivity analysis was performed using a modified continuous PSQI score that excluded the habitual sleep-efficiency component (Component 4). In the adjusted linear regression model, each one-hour delay in bedtime was associated with a 0.33-point higher modified PSQI score (β = 0.327, 95% CI: 0.165–0.490, *p* < 0.001). Thus, the association between later bedtime and poorer sleep quality remained evident after excluding the PSQI component partly derived from sleep-timing information.

As a further sensitivity analysis, the adjusted model was repeated among participants with a PSQI sleep-duration component score of 0, corresponding to at least 7 h of self-reported actual sleep (*n* = 104). Within this subgroup, each one-hour delay in bedtime remained significantly associated with higher odds of poor sleep quality (OR = 1.49, 95% CI: 1.16–1.91, *p* = 0.002). Thus, the association between later bedtime and poor sleep quality remained evident among participants reporting at least 7 h of actual sleep.

## 4. Discussion

This study found that poor sleep quality was common among university esports players, and that habitual bedtime was associated with sleep quality after adjustment for the covariates included in the model. Poor sleep quality was identified in 50.6% of participants; the odds of poor sleep quality were higher among those reporting a chronic illness, and each one-hour delay in bedtime was associated with a 33% increase in the odds of poor sleep quality after adjustment for the other variables included in the model. When bedtime, wake-up time, and mid-sleep time were compared in separate models built with the same covariates, the bedtime model showed the lowest AIC and BIC along with the highest AUC and Nagelkerke R^2^. The association between later bedtime and poor sleep quality remained evident in circular analyses accounting for the cyclical structure of clock-time data, as well as in a sensitivity analysis restricted to participants reporting at least 7 h of actual sleep per night. These findings suggest that, alongside sleep duration, sleep timing also deserves attention when considering the sleep health of university esports players.

### 4.1. Prevalence of Poor Sleep Quality

The rate of poor sleep quality found in this study, 50.6%, is broadly consistent with prior literature reporting that sleep problems among esports players are at a level that should not be underestimated [9,13,14,15,16,21,22,23]. That said, reported rates vary across studies depending on competition level, game genre, country, data collection period, and the measurement approach used. Monma and colleagues reported poor subjective sleep quality in 43.3% of professional and amateur esports players [22]. A recent Healthcare study examining elite esports competitors in Japan found a rate of 38.5% [23]. Smaller studies focused on professional players have likewise reported delayed sleep timing, poor subjective sleep quality, prolonged sleep onset latency, and low sleep efficiency [14,15,16,21]. The relatively high rate observed in the present study may relate to the sample being composed of university students, who may experience academic and social time constraints alongside their esports activities. Even so, direct prevalence comparisons across studies should be made cautiously given differences in sampling and measurement.

### 4.2. Sleep Timing and Sleep Quality

Sleep timing in this sample was notably delayed: median bedtime was 03:00, median mid-sleep time was 06:30, and only 14.6% of participants reported going to bed at midnight or earlier. Median bedtime among participants with poor sleep quality was roughly 90 min later than among those with good sleep quality. This finding aligns with earlier studies showing delayed sleep onset and wake times among professional esports players [14,15,16,21]. Training sessions and matches extending into the evening or night, interaction with players across different time zones, cognitive and emotional arousal after gaming, screen light exposure, and caffeine use are among the factors that may delay bedtime [7,8,9]. As none of these mechanisms were directly measured in the present study, however, they should be regarded not as explanations for the observed association but as possible pathways to be tested in future research.

The finding that each one-hour delay in bedtime was associated with a 33% increase in the odds of poor sleep quality suggests that sleep timing may provide relevant information when assessing sleep health in this population. This is consistent with systematic review evidence linking later and more irregular sleep timing to adverse health outcomes in adults [4]. Going to bed late may widen the mismatch between biological sleep propensity and social obligations, shorten the wind-down period before sleep, and reduce the sleep opportunity available on days with fixed morning schedules. Because of the study’s cross-sectional design, however, it cannot be determined whether late bedtime leads to poor sleep quality, poor sleep quality delays bedtime, or both arise from shared underlying factors such as chronotype, psychological stress, academic schedules, or gaming patterns.

Circular analyses showed that the association was not dependent on the linear recoding used to address the artificial clock-time discontinuity at midnight. The circular mean bedtimes of the good and poor sleep quality groups were 01:59 and 03:27, respectively, and the difference between them was significant; the association between circular bedtime and continuous PSQI score was also confirmed. The variance explained by the circular association, however, was limited (R^2^ = 0.093). Although bedtime was associated with sleep quality in this sample, the limited variance explained by the circular association indicates that bedtime represents only one of multiple factors associated with sleep quality. Sleep quality is a multidimensional construct associated with numerous factors, including psychological state, chronotype, academic load, sleep environment, substance use, and individual health characteristics [1,2].

The more favorable numerical model performance indices observed for the bedtime model relative to the wake-up time and mid-sleep time models suggest that bedtime may capture relevant variation in sleep quality in this sample. Wake time may be more heavily constrained by class schedules and daily obligations, while mid-sleep time is a composite measure reflecting both bedtime and wake time, and indirectly, sleep duration as well. No formal test was conducted to compare the AUC differences between models, however, and the models were not validated in an independent sample. The result should therefore be interpreted not as bedtime being a definitively superior predictor, but as offering the most favorable fit indices among the three indicators examined. ORs quantify associations, whereas AUC, calibration, and Nagelkerke R^2^ reflect apparent in-sample model performance rather than causality or validated predictive utility.

An important methodological consideration is the potential measurement coupling between habitual bedtime and the PSQI global score, because bedtime and getting-up time contribute to the calculation of the habitual sleep-efficiency component (Component 4). Thus, part of the observed association could theoretically reflect this shared measurement structure. To address this issue, we conducted a sensitivity analysis using a modified continuous PSQI score excluding Component 4. Later bedtime remained associated with a higher modified PSQI score after adjustment for the same covariates (β = 0.327, 95% CI: 0.165–0.490, *p* < 0.001). This finding supports the robustness of the observed association to the removal of the mechanically related component, although it does not eliminate the limitations inherent in deriving both sleep timing and sleep quality from the same self-report instrument.

The association between bedtime and poor sleep quality was also observed in the subgroup reporting at least 7 h of actual sleep per night. In this subgroup, each one-hour delay in bedtime was associated with a 49% increase in the odds of poor sleep quality. This finding is consistent with a multidimensional approach to sleep health, in which adequate self-reported sleep duration alone may not fully characterize sleep health and timing, regularity, continuity, and daytime functioning should also be considered [1,2]. In an additional sensitivity analysis, further adjustment for smoking and alcohol use yielded a nearly unchanged estimate (OR = 1.32, 95% CI: 1.12–1.56), indicating that the association was not materially altered by these measured factors. Because sleep duration and timing were derived from the same self-report instrument, however, this sensitivity analysis should not be taken as equivalent to objectively verified adequate sleep duration.

### 4.3. Chronic Illness, Diet, and Gaming Behavior

The higher odds of poor sleep quality among participants reporting a chronic illness is consistent with the close relationship between sleep health and general health [1,2].

Symptoms accompanying chronic illness, pain, medication use, or psychological burden may adversely affect sleep quality; conversely, persistent sleep problems may also be linked to a range of health outcomes. Chronic illness status was self-reported, however, and illness type, duration, severity, and medication use were not recorded. In addition, the number of participants reporting a chronic illness was small (*n* = 23), and the confidence interval for the adjusted OR was wide. The magnitude of this association should therefore be interpreted cautiously and re-examined in larger samples with verified clinical information.

Eating after 21:00 was more common in the poor sleep quality group but did not show an independent association once bedtime and the other covariates were accounted for. This attenuation may reflect overlap between late eating and late bedtime as features of the same daily schedule. Similarly, MEDAS score was not independently associated with poor sleep quality. These findings should not be interpreted as diet being irrelevant to sleep; a single screening score may not fully capture meal timing, energy content, caffeine intake, or short-term dietary changes. Future studies should assess diet quality and meal timing separately and with repeated measures.

Daily gaming duration also did not differ between the good and poor sleep quality groups and showed no independent association in the adjusted model. This is consistent with findings from Kidcaff and colleagues, who found no significant correlation between total gaming duration and sleep quality at the overall sample level, but reported associations that varied by competition level and weekday gaming patterns [12]. Rather than total duration, the time of day gaming occurs, the length of uninterrupted sessions, training intensity, competition-related stress, and symptoms of problematic gaming may be more relevant to sleep quality [9,12,13]. Since none of these characteristics were measured in the present study, the null finding for gaming duration should not be generalized to suggest that esports participation is unrelated to sleep.

### 4.4. Practical Implications and Future Research

These findings suggest that sleep assessment among university esports players should not be limited to asking about total sleep duration alone. Habitual bedtime, sleep regularity, the timing of gaming or training sessions, and daytime functioning could also be considered in sleep assessments. Future intervention studies could examine whether reducing late-night gaming or training sessions, allowing a wind-down period between the end of gaming and bedtime, providing education on caffeine and screen use, and supporting regular sleep opportunities compatible with academic schedules are associated with improvements in sleep-related outcomes [8]. These approaches should be regarded as hypotheses for future research rather than as interventions supported by the present cross-sectional findings. Importantly, the observed cross-sectional association does not permit the conclusion that going to bed earlier would improve sleep quality. A brief sleep education intervention combining individual counseling and biofeedback, delivered to professional esports players, produced a modest improvement in insomnia severity but no clear benefit for mood or cognitive performance [24]. This suggests that tailored approaches, with referral to specialist support when needed, may warrant investigation rather than uniform, low-intensity interventions.

Future research should follow university esports players across different periods, jointly assessing class and exam schedules, weekday–weekend differences, chronotype, social jetlag, light exposure, caffeine and energy drink consumption, psychological stress, symptoms of problematic gaming, and game genre. Using repeated, objective measures such as actigraphy, alongside sleep diaries, would allow habitual bedtime to be verified and within-person variation to be assessed. In addition, interventions targeting earlier or more regular bedtimes should be evaluated for their effects on sleep quality, daytime functioning, and gaming performance using randomized or strong quasi-experimental designs.

### 4.5. Strengths and Limitations

Strengths of this study include its direct focus on university esports players with previous competition experience, its modeling of bedtime as a continuous variable reflecting hour-by-hour variation rather than arbitrary categories, and its comparison of bedtime, wake-up time, and mid-sleep time in models built with the same covariates. Re-examining clock times using circular statistics showed that the primary finding did not depend on the choice of linear coding. The association was also observed among participants reporting at least 7 h of actual sleep, indicating that the association remained evident in this duration-restricted subgroup. Apparent in-sample model discrimination and calibration were acceptable, and multicollinearity indicators were low.

Several limitations should nonetheless be considered. First, the cross-sectional design does not allow temporal sequencing or causality to be established. Second, convenience sampling through university esports-related settings in Istanbul may introduce selection bias, and the predominantly male composition of the sample further limits generalizability; therefore, the findings cannot be generalized without further evidence to female players, professional teams, recreational gamers, other age groups, or other geographical settings. Third, sleep quality and timing were self-reported; the PSQI is not a clinical diagnostic tool, and bedtime may not fully correspond to sleep onset. Weekday and weekend timing were not assessed separately, and regularity and social jetlag were not measured. Fourth, excluding individuals with a self-reported psychiatric diagnosis may have reduced the representativeness of the sample and may limit generalizability to university esports players with psychiatric conditions. The lack of measurement of potential confounders such as chronotype, stress, depressive symptoms, caffeine and energy drink use, nighttime screen exposure, medication use, physical activity, game genre, and training hours increases the likelihood of residual confounding. Fifth, chronic illness was assessed by self-report and as a binary variable, and the small number of participants in this group limited the precision of the association estimate. Finally, model performance was evaluated within the same sample; no internal or external validation was performed, and no formal comparison was made between the AUCs of the timing models. Model metrics should therefore be considered exploratory.

In conclusion, poor sleep quality was common among university esports players, and later bedtime was consistently associated with poor sleep quality, including among players reporting at least 7 h of actual sleep per night. These findings suggest that habitual bedtime deserves consideration alongside sleep duration when assessing sleep health in university esports players. However, longitudinal and experimental studies are needed to determine the direction of the association and whether modifying bedtime improves sleep quality.

## Figures and Tables

**Table 1:** Characteristics of esports players (*n* = 164).

Variable	Value
***Sociodemographic and anthropometric variables***	
Age (years), mean ± SD	22.52 ± 3.13
Sex, *n* (%)	Male: 147 (89.63); Female: 17 (10.37)
BMI (kg/m^2^), mean ± SD	24.44 ± 4.49
BMI classification, *n* (%)	Underweight: 10 (6.10); Normal: 85 (51.83); Overweight: 54 (32.93); Obese: 15 (9.15)
Chronic disease, *n* (%)	Yes: 23 (14.02); No: 141 (85.98)
Smoking, *n* (%)	Yes: 82 (50.00); No: 82 (50.00)
Alcohol consumption, *n* (%)	Yes: 101 (61.59); No: 63 (38.41)
***Gaming-related variables***	
Years in esports, median (IQR)	5.00 (3.00–7.00)
Weekly gaming days, median (IQR)	5.00 (3.00–7.00)
Daily gaming duration (hours), median (IQR)	5.00 (4.00–8.00)
Competitions won, median (IQR)	1.50 (0.00–4.00)
***Dietary habits***	
Number of main meals, median (IQR)	2.00 (2.00–3.00)
Number of snacks, median (IQR)	1.00 (1.00–2.00)
Breakfast skipping, *n* (%)	112 (68.29)
First meal before 09:00, *n* (%)	50 (30.49)
Eating after 21:00, *n* (%)	108 (65.85)
Supplement use (any dietary supplement), *n* (%)	60 (36.59)
Daily water intake, *n* (%)	Less than 2 L: 61 (37.20); 2 L and more: 103 (62.80)
MEDAS score, mean ± SD	6.54 ± 2.40
MEDAS classification, *n* (%)	Non-adherent: 87 (53.05); Moderately adherent/Acceptable adherence: 48 (29.27); Adherent: 29 (17.68)
***Sleep variables***	
PSQI score, mean ± SD	5.88 ± 2.69
Good sleep quality, *n* (%)	81 (49.39)
Poor sleep quality, *n* (%)	83 (50.61)
Bedtime, median (IQR)	03:00 (01:00–04:30)
Wake-up time, median (IQR)	10:30 (09:00–12:00)
Mid-sleep time, median (IQR)	06:30 (05:00–08:30)
Bedtime-to-wake-up interval (hours), median (IQR)	8.00 (7.00–9.00)

Continuous variables are presented as mean ± SD or median (IQR), and categorical variables as *n* (%). BMI: Body Mass Index; IQR: Interquartile Range; MEDAS: Mediterranean Diet Adherence Screener; PSQI: Pittsburgh Sleep Quality Index.

**Table 2:** Comparison of esports players according to sleep quality.

Variable	Good Sleep (*n* = 81)	Poor Sleep (*n* = 83)	*p*	Effect Size
Age, median (IQR)	22.00 (21.00–24.00)	22.00 (21.00–23.00)	0.388	r = 0.067
Sex (male), *n* (%)	76 (93.83)	71 (85.54)	0.082	Cramer’s V = 0.136
BMI, median (IQR)	24.86 (21.80–26.85)	23.51 (20.88–26.35)	0.119	r = 0.122
Chronic disease (yes), *n* (%)	4 (4.94)	19 (22.89)	<0.001 *	Cramer’s V = 0.258
MEDAS score, median (IQR)	7.00 (6.00–8.00)	6.00 (5.00–8.00)	0.166	r = 0.107
Bedtime, median (IQR)	02:00 (00:30–03:00)	03:30 (02:00–05:00)	<0.001 *	r = 0.352
Bedtime-to-wake-up interval (h)	8.50 (7.50–9.00)	7.50 (6.00–9.00)	0.003 *	r = 0.229
Breakfast skipping, *n* (%)	51 (62.96)	61 (73.49)	0.147	Cramer’s V = 0.113
First meal before 09:00, *n* (%)	29 (35.80)	21 (25.30)	0.144	Cramer’s V = 0.114
Eating after 21:00, *n* (%)	47 (58.02)	61 (73.49)	0.037 *	Cramer’s V = 0.163
Main meals, median (IQR)	2.00 (2.00–3.00)	2.00 (2.00–3.00)	0.933	r = 0.006
Snacks, median (IQR)	1.00 (1.00–2.00)	1.00 (1.00–2.00)	0.062	r = 0.139
Daily gaming hours, median (IQR)	5.00 (4.00–8.00)	5.00 (4.00–8.00)	0.915	r = 0.008

* *p* < 0.05. Group comparisons are presented as median (IQR) for continuous variables and *n* (%) for categorical variables. Effect sizes are reported as rank-biserial correlation (r) for Mann–Whitney U tests and Cramer’s V for categorical variables. BMI: Body Mass Index; MEDAS: Mediterranean Diet Adherence Screener.

**Table 3:** Univariable and multivariable logistic regression analyses for factors associated with poor sleep quality (PSQI > 5).

Variable	Crude OR	Adjusted OR	95% CI (Adjusted)	*p* (Adjusted)
Age	0.96	1.02	0.91–1.13	0.796
Sex (male)	0.39	0.44	0.12–1.60	0.213
BMI	0.94	0.95	0.87–1.03	0.211
Chronic disease (yes)	5.71	5.60	1.72–18.28	0.004 *
MEDAS score	0.90	0.96	0.83–1.11	0.540
Bedtime (per hour)	1.29	1.33	1.13–1.57	<0.001 *
Eating after 21:00 (yes)	2.01	1.40	0.67–2.89	0.370
Daily gaming duration (hours)	1.01	0.95	0.84–1.07	0.387

Crude ORs are from univariable logistic regression models. Adjusted ORs are from one multivariable logistic regression model including all variables shown in the table. * *p* < 0.05. Outcome variable is poor sleep quality (PSQI > 5). Bedtime is modeled as a continuous variable on the recoded late-night scale (odds ratio per additional hour of delay). OR: odds ratio; CI: confidence interval; BMI: Body Mass Index; MEDAS: Mediterranean Diet Adherence Screener.

**Table 4:** Comparison of bedtime, wake-up time, and mid-sleep time in separate adjusted logistic regression models of poor sleep quality (PSQI > 5).

Timing Metric	Adjusted OR (per Hour)	95% CI	*p*	AUC	Nagelkerke R^2^	AIC/BIC
Bedtime	1.33	1.13–1.57	<0.001 *	0.743	0.245	212.0/239.9
Wake-up time	1.08	0.94–1.26	0.286	0.686	0.159	224.5/252.4
Mid-sleep time	1.25	1.06–1.48	0.010 *	0.714	0.200	218.6/246.5

AUC: area under the receiver operating characteristic curve; CI: confidence interval; OR: odds ratio. * *p* < 0.05. All three models include the same covariates (age, sex, BMI, chronic disease, MEDAS score, eating after 21:00, daily gaming hours) and differ only in which continuous sleep-timing variable is entered.

## Data Availability

The datasets used and/or analyzed during the current study are not publicly available due to privacy and ethical considerations but are available from the corresponding author on reasonable request.

## Supplementary Materials

The following supporting information can be downloaded at https://www.mdpi.com/article/10.3390/healthcare14183103/s1. Figure S1: Frequency distribution of the 10 most frequently reported esports game titles among participants in the analytic sample.

## Abbreviations

The following abbreviations are used in this manuscript:

BMI	Body Mass Index
CI	Confidence Interval
IQR	Interquartile Range
MEDAS	Mediterranean Diet Adherence Screener
OR	Odds Ratio
PSQI	Pittsburgh Sleep Quality Index
SD	Standard Deviation
VIF	Variance Inflation Factor
