# Supplementary material for: Late Bedtime and Poor Sleep Quality Among University Esports Players: A Cross-Sectional Study
Source: Healthcare (Basel). 2026 Sep 20;14(18):3103. doi: 10.3390/healthcare14183103 (PMC13606402; doi:10.3390/healthcare14183103)
Supplement: Supplementary file 1 [file healthcare-14-03103-s001.zip › healthcare-4523993-supplementary.pdf]

Supplementary Materials

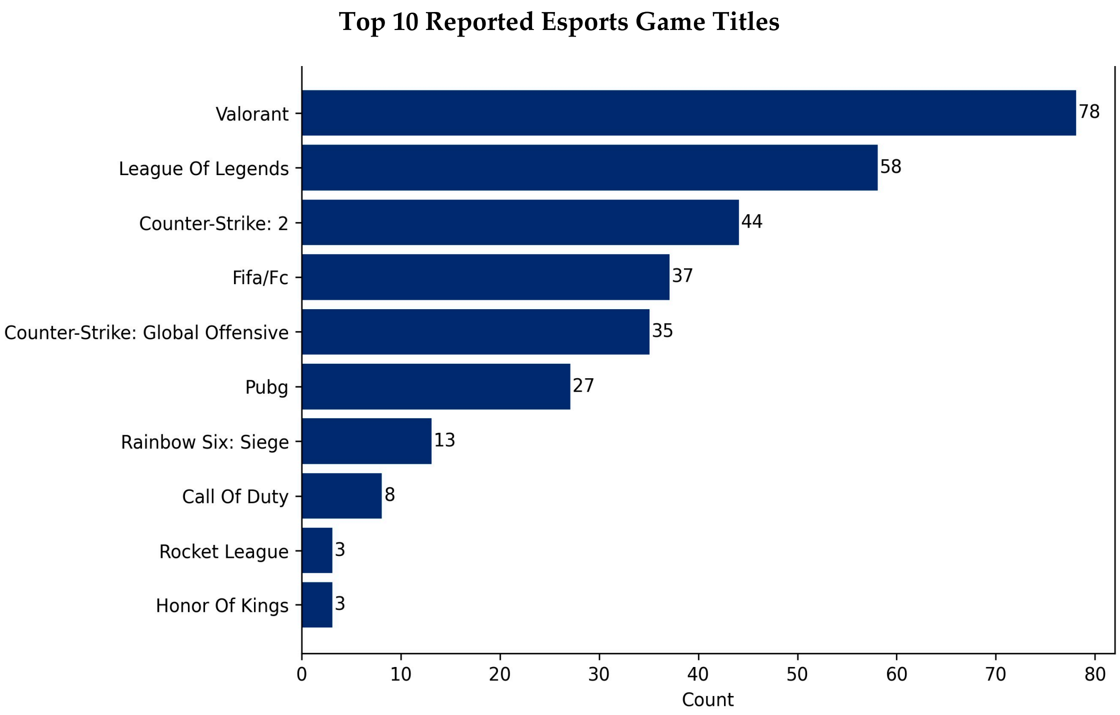

Figure S1. Frequency distribution of the 10 most frequently reported esports game titles among participants in the analytic sample.
